# Genital *Brucella suis* Biovar 2 Infection of Wild Boar (*Sus scrofa*) Hunted in Tuscany (Italy)

**DOI:** 10.3390/microorganisms9030582

**Published:** 2021-03-12

**Authors:** Giovanni Cilia, Filippo Fratini, Barbara Turchi, Marta Angelini, Domenico Cerri, Fabrizio Bertelloni

**Affiliations:** Department of Veterinary Science, University of Pisa, Viale delle Piagge 2, 56124 Pisa, Italy; filippo.fratini@unipi.it (F.F.); barbara.turchi@unipi.it (B.T.); marta94.ang@gmail.com (M.A.); domenico.cerri@unipi.it (D.C.); fabrizio.bertelloni@unipi.it (F.B.)

**Keywords:** *Brucella suis* biovar 2, wild boar, surveillance, epidemiology, reproductive system

## Abstract

Brucellosis is a zoonosis caused by different *Brucella* species. Wild boar (*Sus scrofa*) could be infected by some species and represents an important reservoir, especially for *B. suis* biovar 2. This study aimed to investigate the prevalence of *Brucella* spp. by serological and molecular assays in wild boar hunted in Tuscany (Italy) during two hunting seasons. From 287 animals, sera, lymph nodes, livers, spleens, and reproductive system organs were collected. Within sera, 16 (5.74%) were positive to both rose bengal test (RBT) and complement fixation test (CFT), with titres ranging from 1:4 to 1:16 (corresponding to 20 and 80 ICFTU/mL, respectively). *Brucella* spp. DNA was detected in four lymph nodes (1.40%), five epididymides (1.74%), and one fetus pool (2.22%). All positive PCR samples belonged to *Brucella suis* biovar 2. The results of this investigation confirmed that wild boar represents a host for *B.* *suis* biovar. 2 and plays an important role in the epidemiology of brucellosis in central Italy. Additionally, epididymis localization confirms the possible venereal transmission.

## 1. Introduction

Wild boar (*Sus scrofa*) is a large mammal that is globally spread, and it is able to colonize different habitats, including suburban and urban areas [1,2]. Wild boar has early puberty, from 5 to 10 months of age. They are characterized by high fertility and a gestation period from 115 to a maximum of 122 days [3,4,5,6]. Usually, the mating season is once a year, and males mate with more than one female [5,6,7]. Piglet births occur usually during the late winter and early spring, with a peak during February or March [7,8,9]. Furthermore, wild boar reproductive parameters are highly influenced by different features, such as habitat, climatic conditions, photoperiods, hunting pressure, and availability of food resources [6,9,10]. In recent years, in Europe, as well as in Italy, the population number constantly increased due to the high adaptability of these animals [1]. For some Italian regions, such as Tuscany, a high wild boar population density was estimated [11] considering the high number of hunted animals [11,12,13,14]. This aspect represents a serious problem for agriculture and public health [15,16]. As for the latter, wild boar could act as a reservoir for different bacterial pathogens, contributing to maintaining and disseminating some important infectious diseases and zoonosis, including brucellosis [17,18,19,20,21,22,23].

The genus Brucella includes 12 species: B. abortus, B. canis, B. ovis, B. suis, B. melitensis, B. neotomae, B. ceti, B. pinnipedialis, B. microti, B. inopinata, B. vulpis, and B. papionis. Among these, B. abortus, some biovars of B. suis, and, rarely, B. melitensis can infect swine, as well as wild boar [24,25]. Recently, B. microti was also isolated from the lymph node of a wild boar in the Czech Republic [26]. In Tuscany, as well as in many parts of Italy, bovine and ovine brucellosis was eradicated from several years [27]. As for B. suis, it was rarely reported in Europe, except for biovar 2, which is widely spread in East Europe and was recently isolated from domestic pigs and wild boar in Italy [28,29,30]. Wild boar represents one of the most important B. suis biovar 2 reservoirs. Furthermore, B. suis biovar 2 infection was recently reported in cows, in which seroconversion was detected without the presence of clinical signs [31,32]; human infections were rarely reported [33].

Brucellosis in swine caused by *B. suis*, especially biovar 2, is usually responsible for systemic infection and causes chronic diseases [24,34]. Bacteremia could persist for several months, and *B. suis* may persist in the uterus, causing chronic metritis [35,36]. Moreover, in males, genital infections seem to be frequent, with a tropism for epididymis. Brucellosis is responsible for reproductive disorders, characterized by abortion, stillbirths, decreased litter size, weak piglets, infertility, orchitis and epididymitis in males, and focal abscess formation [34,35,37]. The localization of *B. suis* in the epididymis could be due to a possible infection through venereal transmission [36].

This study aimed to assess *Brucella* spp. diffusion in wild boar hunted in the Tuscany region (Italy) to delineate the risk of spreading and possible transmission to animals and humans. For these purposes, serological and molecular assays were employed. Moreover, genital localization in male and female organs, including uterus, placenta and fetuses of pregnant animals, were analyzed to investigate the possibility of venereal and vertical transmissions.

## 2. Materials and Methods

### 2.1. Sample Collection

Over two consecutive hunting seasons (from November 2018 to January 2019 and from November 2019 to January 2020), serum and several organs were sampled from hunted wild boar. Blood samples were collected through an ocular puncture to obtain sera [38]. Moreover, from the same animals, mesenteric lymph nodes, spleen, liver, and reproductive system were collected. Regarding genital organs, testicles, epididymides, and uteri were sampled, as well as placentas and fetuses from pregnant wild boar. Organs (lungs, stomach, liver, and kidneys) obtained from all fetuses collected from the same pregnant animal were pooled and considered as a single sample. The sex and age class of each specimen were determined. The age class determination was performed by assessing the degree of tooth eruption and the wear and tear of teeth of the lower jaw, classifying three age groups: young (under 12 months), subadult (between 12 and 24 months), and adult (over 24 months) [39]. All animals were hunted in the Tuscany region during authorized hunting seasons, following the regional hunting law (Regolamento di attuazione della Legge Regionale 12 gennaio 1994, n. 3 D.P.G.R. 48/R/2017). Because sampling activities were performed in collaboration with hunter companies, the final number of samples was not predicted beforehand. No animals were sacrificed specifically for this study.

### 2.2. Serological Investigations

Within three hours, blood samples were transported in refrigerated conditions to the Laboratory of Infectious Diseases of the Department of Veterinary Science, University of Pisa (Italy), and obtained sera were stored at −20 °C until analysis [38].

A rose bengal test (RBT) and complement fixation test (CFT) were employed to detect anti-brucella antibodies. RBT and CFT were performed as described by the World Organization for Animal Health (OIE) [40]; antigens used in both tests were obtained from the Istituto Zooprofilattico Sperimentale dell′Abruzzo e del Molise G. Caporale, Teramo. Considering the intrinsic limitation of the employed methods [40], only samples showing a positive reaction to both tests were considered as positive. As a positive and negative control, positive pig serum (provided by Istituto Zooprofilattico Sperimentale dell′Abruzzo e del Molise G. Caporale, Teramo) and sterilized saline water were employed, respectively.

### 2.3. Molecular Investigations

DNA was extracted from 25 µg of each tissue sample (lymph node, spleen, liver, and reproductive organs) using the Quick-DNA Plus Kits (Zymo Research, Irvine, CA, USA), following the manufacturers’ instructions. All the samples were tested by real-time PCR to identify the *Brucella* genus-specific target (*bcsp31* gene) using a Rotorgene Corbett 6000 (Corbett Research, Sidney, Australia) [41]. Subsequently, only *Brucella*-positive samples, were subjected to further real-time PCR assays to discriminate *B. abortus, B. suis, B. melitentis*, and *B. ovis* [42,43]. A total reaction volume of 15 μL was prepared using 2× QuantiTect Probe PCR Master Mix (Qiagen, Hilden, Germany), 2 μM of each primer, 500 nM of each probe, and 3 μL of DNA. Finally, another real-time PCR set was performed to identify the *Brucella suis* biovars [44]. The amplification of the target gene was performed using the HotStarTaq Master Mix Kit (Qiagen, Hilden, Germany) [44]. As a negative control, sterilized saline water was used. Amplicons were further sequenced (BMR Genomics, Padova, Italy) using the same amplification primer sets and analyzed using BioEdit Software [45]. Phylogenetic analysis was performed by the maximum likelihood method based on the Tamura-Nei model using MEGA 10 software [46].

### 2.4. Statistical Analysis

Data were analyzed with the chi-square (*X*^2^) test. The statistical test was used to evaluate serological- and molecular-positive rates in relation to sex (male or female), age class (young, subadult, or adult), province (Pisa, Lucca, Livorno, Grosseto or Siena), and hunting season (2018/2019 or 2019/2020). The statistical significance threshold was set at a *p*-value ≤ 0.05 [47].

## 3. Results

**Figure 1:**
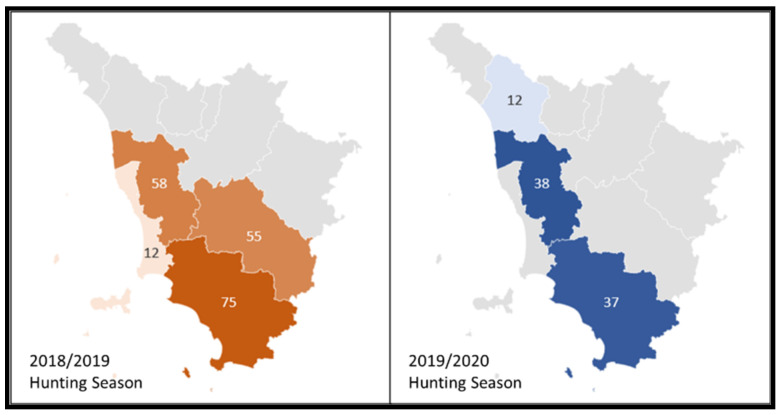
Geographical distribution of the sampling area included in the study (Tuscany region, Italy). The number of sampled hunted wild boar per province is indicated for hunting seasons (adapted by Cilia et al. [21]). In orange, hunting season 2018/2019; in blue, hunting season 2019/2020.

Serum, lymph nodes, liver, spleen, and reproductive organs were collected from a total of 287 hunted wild boar: 118 males (86 and 32 from 2018/2019 and 2019/2020 hunting seasons, respectively) and 169 females (115 and 54 from 2018/2019 and 2019/2020 hunting seasons, respectively). Considering the sampling area, 200 wild boar were sampled during the 2018/2019 period: 75 from Grosseto province, 58 from Pisa province, 55 from Siena province, and 12 from Livorno province (Figure 1). In addition, 87 animals were investigated during the 2019/2020 period: 38, 37, and 12 wild boar were sampled from Pisa, Grosseto, and Lucca provinces, respectively (Figure 1).

Moreover, 110 testicles and epididymides were collected from males (77 in 2018/2019 and 33 in 2019/2020); 37 uteri from females (36 in 2018/2019 and 1 in 2019/2020); and 45 uteri, placentae, and fetuses from pregnant females (34 in 2018/2019 and 11 in 2019/2020).

Results on the distribution of positive sera and organs for *Brucella* in relation to hunting season, province, sex, and age class are reported in Table 1. In total, 26 wild boar specimens scored positive for *Brucella* spp. infection by serological or molecular analysis or both.

### 3.1. Serological Investigations

The results of this investigation showed that 16 out of 287 sera (5.57%) scored positive for both serological assays. Titres ranging from 1:4 to 1:16 (corresponding to 20 and 80 international complement fixation test units per ml—ICFTU/mL, respectively) were determined by CFT. In relation to hunting season, 11 out of 200 (5.5%) and 6 out of 87 (6.90%) scored positive in 2018/2019 and 2019/2020, respectively (Table 2). No statistical differences (*p* > 0.05) were detected for the serological positivity considering all parameters. Because Pisa and Grosseto are the only two provinces investigated during the two continuous hunting seasons, and comparing the same issues, no statistical differences (*p* > 0.05) were highlighted.

### 3.2. Molecular Investigations

Concerning molecular analysis, *Brucella* spp. DNA was detected in lymph nodes, epididymides, and fetuses, although no liver, spleen, testicle, uterus or placenta samples scored positive. Overall, 10 out of 287 (3.48%) wild boar scored positive through real-time PCR. All of them scored negative in serological assays.

Specifically, 4 out of 287 (1.40%) lymph nodes (all sampled in the 2018/2019 hunting season, 2.00%) were positive (Table 2). In 5 epididymides (1.74%), *Brucella* spp. DNA was found, 4 (2.00%) and 1 (1.15%) collected during the 2018/2019 and 2019/2020 hunting seasons, respectively (Table 2). Moreover, only 1 fetus pool out of 45 (2.22%) from an adult pregnant female hunted in 2019/2020 was positive. For lymph nodes, epididymides, and fetuses, no statistical differences (*p* > 0.05) were reported considering hunting seasons, provinces, and wild boar sex and age class, as well as when comparing Pisa and Grosseto during the two different hunting seasons.

**Figure 2:**
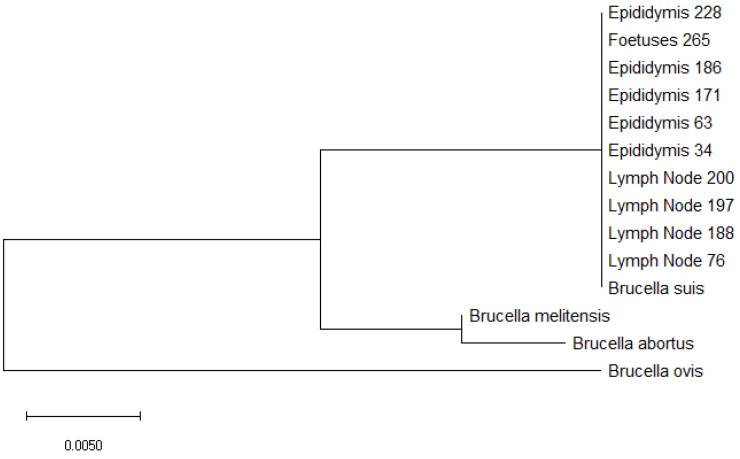
Molecular phylogenetic analysis for 16s rRNA gene of positive lymph nodes and epididymides with *Brucella* *suis*, *Brucella* *melitensis*, *Brucella abortus*, and *Brucella ovis* by the maximum likelihood method based on the Tamura–Nei model. The branch lengths of the tree measured the number of substitutions per site. The analysis involved 14 nucleotide sequences. There was a total of 534 positions in the final dataset.

All PCR-positive samples showed the presence of *Brucella* DNA belonging to *Brucella suis*. This was confirmed by phylogenetic analysis of sequences (Figure 2). Moreover, all *Brucella suis* belonged to biovar 2.

## 4. Discussion

The present investigation carried out on wild boar hunted in the Tuscany region (central Italy) reports seroprevalence for *Brucella* spp. and the identification of *Brucella suis* biovar 2 in lymph nodes, epididymides, and fetuses. Organs and sera were sampled during two consecutive hunting seasons (2018/2019 and 2019/2020), and no statistical differences were highlighted between wild boar sex, age class, provinces, and hunting seasons.

A previous study conducted from 1997 to 2000 in the same area did not report seropositivity to *Brucella* spp. in free-ranging wild boar [48], whereas, during the 2017/2018 season, serological positivity was highlighted in 4.01% of specimens [14], which is consistent with results obtained in this investigation (5.75% of seropositivity). These data suggest an increase in wild swine brucellosis in Tuscany over the last two decades. The percentages of positive wild boar sera and lymph nodes detected in this survey for *Brucella* spp. suggested a low infection rate of brucellosis among the wild boar population in Tuscany, as already demonstrated in other Italian regions [29,49,50,51,52,53,54] and Europe [23,55,56,57,58,59,60]. Traditional serological assays, RBT and CFT, do not allow the identification of *Brucella* species involved in the infection. However, because Tuscany and adjacent regions are currently free from bovine, ovine, and caprine brucellosis [27], and considering that in Central Italy, *Brucella suis* biovar 2 was recently isolated from wild boar [29] and pigs [28], it is possible to theorize that the detected serological positivity could be linked to an infection by the same biovar. Moreover, this hypothesis seems to be confirmed by *Brucella suis* biovar 2 identification in sampled organs by molecular methods.

Our results show no correlation between serological and molecular results. Few specific studies on wild boar were carried out regarding this issue, and most of them were performed on naturally infected animals. Regarding the real-time PCR protocol employed, it was reported to have great sensitivity for the pathogen. This assay can detect a low concentration of *Brucella* DNA, corresponding to 0.25 pg; assuming that 10–15 fg of DNA is equivalent to 1 genome, 0.25 pg of DNA corresponds to about 16–25 genome copies [41,42]. On the other hand, RBT and CFT in swine are not free from false-positive reactions. Indeed, seronegativity of infected animals was previously reported by other authors and was attributed mainly to an intrinsic limit of the employed test and to the fact that serological tests are developed and standardized for domestic animals and not for feral swine [61]. In our case, some more aspects could be considered. Molecular analyses showed the presence of *B. suis* biovar 2 in lymph nodes of four seronegative animals; in particular, two of them were young, one was a subadult, and one an adult (animals older than about two years). In these animals, the occurrence of a possible congenital infection could be supposed. This condition is well described for bovine where “latent”, “symptomless”, or “chronic” serologically negative carriers are well described [62]. The detection of *B. suis* biovar 2 DNA in one fetus pool highlights the possibility of vertical transmission in this animal species. Five wild boar scored positive at the epididymis level only, without serological reactions. This could be linked to more sensitivity of molecular tests compared to serological tests, as suggested and shown by other authors [63]. Furthermore, there is the possibility of a restricted localization in the epididymis, causing low stimulation of the immune system and, consequently, no detectable antibodies. This could be the consequence of venereal transmission of the disease linked to a sexual transmission cycle among the wild boar population. This hypothesis could be supported by the fact that in these animals, *Brucella* DNA was detected only in the genital tract and not in lymph nodes. Finally, all serologically positive animals were PCR-negative, which could appear unusual. However, some authors have reported the impossibility of detecting *Brucella* in wild or domestic animals scored positive for serological examination, in some cases, even during experimental infections [64,65,66]. Furthermore, the negative results of PCR examinations on all livers and spleens could suggest chronic infection, a condition probably characterized by the presence of antibodies, at low titer in many cases, and associated with a low number of non-detectable bacteria in “latency” organs.

Brucellosis is characterized by genital tropism, causing orchitis, epididymitis, and infertility in males and abortion and sterility in females [67,68,69]. Moreover, *Brucella suis* has been demonstrated to be able to infect the reproductive system, especially in males [24,70]. In this investigation, *B. suis* biovar 2 has been detected in wild boar epididymides and fetuses. To the best of the authors’ knowledge, this is the first evidence of *Brucella suis* biovar 2-specific localization in the epididymis of wild boar. Indeed, the obtained results show that the epididymis, and not testicles, seems to be the target organ of localization for *B. suis* biovar 2 in this animal species. This finding is consistent with the localization of other *Brucella* species, for example, *B. ovis* in rams [71]. The tropisms of *Brucella* spp. for epididymides and fetuses, instead of other genital organs, is related to the abundant presence of erythritol and fructose in these organs [72,73,74]. Fructose serves as the primary source of energy for the bacteria [75], and the high concentration of erythritol in fetal fluid, epididymis, and semen is strongly associated with genital brucellosis [76,77,78]. To the best of the authors’ knowledge, no data are available in the literature about the exact localization of *B. suis* biovar 2 in the male reproductive tract of wild boar. Many studies investigated the presence of *B. suis* biovar 2 in the asymptomatic boar genital tract without specifying the exact organ, or sometimes reporting negative detection in testicles [50,56]. No macroscopic lesions were detected in any male genital organs collected during this survey. Epididymis localization in presumptive asymptomatic animals could induce a constant release of *Brucella* through the semen and consequently during coitus. The last hypothesis paves the way for new, interesting epidemiological considerations linked to wild boar reproductive behavior. Adult males are solitary animals living alone and searching for female groups during the reproductive season, sometimes crossing long distances, driving away other young or subadult sexually mature males and adult contenders, and eventually copulating with as many receptive sows as possible [5,6,7]. In this way, positive males could contribute to the spreading of *B. suis* biovar 2 by venereal transmission. The roaming of adult or subadult males, especially during reproductive seasons, could contribute to the diffusion of the disease. Indeed, regarding the Tuscany region, *Brucella* infection was previously reported in wild boar and pigs only in the south of the region [14,29,51,79,80], whereas the present data suggest a diffusion of the disease in all investigated provinces without statistical differences.

## 5. Conclusions

This investigation evaluated the presence of *Brucella* in wild boar through serological and molecular assays. As expected, due to brucellosis eradication in central Italy and species-specific association, *Brucella* *suis* resulted in being the only detected species.

The obtained data show the presence of *Brucella suis* biovar 2 in wild boar lymph nodes and, for the first time, in epididymides and fetuses. Because it is a neglected or underestimated issue, the impact and epidemiological role of *Brucella suis* infection on the reproductive system and, consequently, in reproductive performances of wild boar could be of interest. The monitoring seems to be of great importance because the infection could also be silent or chronic, increasing the possibility of spreading of disease among wild boar populations. Moreover, the infection in the reproductive system of wild boar could represent a serious hazard for swine, especially when semi-extensive breeding is adopted, as in several central Italy areas where breeding between domestic and wild swine is common. Further investigations should be performed to understand the prevalence of *Brucella suis* in the reproductive system and fetuses and the possible implication of venereal and vertical transmission among the wild boar population. Furthermore, other wild or domestic animals, such as hares, sharing the same environment should be included in a more complete monitoring program.

## Figures and Tables

**Table 1:** Distribution of positive sera and organs for pathogenic *Brucella* spp. in relation to hunting season, province, sex, and age class.

Hunting Season	Province	Sex	Age Class	Examined Wild Boar	Positive Sera (%)	PCR-Positive Lymph Nodes (%)	PCR-Positive Epididymides (%)	PCR-Positive Fetuses (%)
2018/2019	Pisa	Male	Adult	9	0	0	0	
(*n* = 200)	(*n* = 58)	(*n* = 30)	Subadult	10	3 (30.0)	0	2 (20.0)	
			Young	11	0	0	0	
		Female	Adult	14	1 (7.14)	0		0
		(*n* = 28)	Subadult	5	0	0		0
			Young	9	2 (22.2)	1 (11.1)		0
	Grosseto	Male	Adult	10	0	0	1 (10.0)	
	(*n* = 75)	(*n* = 29)	Subadult	5	0	1 (20.0)	0	
			Young	14	0	1 (7.14)	0	
		Female	Adult	22	0	0		0
		(*n* = 46)	Subadult	5	1 (20.0)	0		0
			Young	19	1 (5.26)	0		0
	Siena	Male	Adult	10	0	0	1 (10.0)	
	(*n* = 55)	(*n* = 22)	Subadult	4	0	0	0	
			Young	8	0	0	0	
		Female	Adult	21	1 (4.76)	0		0
		(*n* = 33)	Subadult	2	0	0		0
			Young	10	0	0		0
	Livorno	Male	Adult	2	0	1 (50.0)	0	
	(*n* = 12)	(*n* = 4)	Subadult	0	0	0	0	
			Young	2	1 (50.0)	0	0	
		Female	Adult	4	0	0		0
		(*n* = 8)	Subadult	0	0	0		0
			Young	4	0	0		0
2019/2020	Pisa	Male	Adult	6	2 (33.4)	0	0	
(*n* = 87)	(*n* = 38)	(*n* = 13)	Subadult	4	0	0	0	
			Young	3	0	0	0	
		Female	Adult	21	1 (4.76)	0		1 (4.76)
		(*n* = 25)	Subadult	1	0	0		0
			Young	3	0	0		0
	Grosseto	Male	Adult	11	1 (9.10)	0	1 (9.10)	
	(*n* = 37)	(*n* = 16)	Subadult	1	0	0	0	
			Young	4	0	0	0	
		Female	Adult	10	1 (20.0)	0		0
		(*n* = 21)	Subadult	5	0	0		0
			Young	6	0	0		0
	Lucca	Male	Adult	1	1 (100)	0	0	
	(*n* = 12)	(*n* = 4)	Subadult	0	0	0	0	
			Young	3	0	0	0	
		Female	Adult	4	0	0		0
		(*n* = 8)	Subadult	0	0	0		0
			Young	4	0	0		0

**Table 2:** *Brucella* spp. positive wild boar specimens in relation to hunting seasons, province, sex, age, serological, and molecular investigations.

Sample	Hunting Season	Province	Sex	Age	Serological		PCR PositiveTissue	Sequencing
					RBT	FdC *		
C14	2018/2019	PI	M	Subadult	+	1:4	None	/
C23	2018/2019	LI	M	Young	+	1:4	None	/
C31	2018/2019	PI	M	Adult	+	1:8	None	/
C33	2018/2019	PI	F	Young	+	1:4	None	/
C34	2018/2019	PI	M	Subadult	-	-	Epididymis	*B. suis* bv. 2
C63	2018/2019	SI	M	Adult	-	-	Epididymis	*B. suis* bv. 2
C76	2018/2019	PI	F	Young	-	-	Lymph nodes	*B. suis* bv. 2
C82	2018/2019	GR	Fe	Subadult	+	1:8	None	*/*
C141	2018/2019	GR	F	Young	+	1:4	None	*/*
C150	2018/2019	PI	M	Subadult	+	1:4	None	*/*
C155	2018/2019	SI	F	Adult	+	1:16	None	*/*
C171	2018/2019	GR	M	Adult	-	-	Epididymis	*B. suis* bv. 2
C172	2018/2019	PI	M	Subadult	+	1:8	None	*/*
C175	2018/2019	PI	M	Young	+	1:4	None	*-*
C186	2018/2019	PI	M	Subadult	-	-	Epididymis	*B. suis* bv. 2
C188	2018/2019	LI	M	Adult	-	-	Lymph nodes	*B. suis* bv. 2
C197	2018/2019	GR	M	Young	-	-	Lymph nodes	*B. suis* bv. 2
C200	2018/2019	GR	M	Subadult	-	-	Lymph nodes	*B. suis* bv. 2
C209	2019/2020	LU	M	Adult	+	1:16	None	*/*
C218	2019/2020	GR	M	Adult	+	1:4	None	*/*
C228	2019/2020	GR	M	Adult	-	-	Epididymis	*B. suis* bv. 2
C242	2019/2020	PI	F	Adult	+	1:4	None	*/*
C251	2019/2020	GR	F	Adult	+	1:8	None	*/*
C259	2019/2020	PI	F	Adult	-	-	Fetuses	*B. suis* bv. 2
C266	2019/2020	PI	M	Adult	+	1:4	None	*/*
C267	2019/2020	PI	M	Adult	+	1:8	None	*-*

Legend: PI = Pisa, LI = Livorno, GR = Grosseto, SI = Siena, LU = Lucca; M = male, F = female; * 1:4 = 20 ICFTU/mL; 1:8 = 40 ICFTU/mL; 1:16 = 80 ICFTU/mL.

## Data Availability

The data presented in this study are available in the article.
